# Effect of malaria on placental volume measured using three-dimensional ultrasound: a pilot study

**DOI:** 10.1186/1475-2875-11-5

**Published:** 2012-01-05

**Authors:** Marcus J Rijken, William E Moroski, Suporn Kiricharoen, Noaeni Karunkonkowit, Gordon Stevenson, Eric O Ohuma, J Alison Noble, Stephen H Kennedy, Rose McGready, Aris T Papageorghiou, François H Nosten

**Affiliations:** 1 Shoklo Malaria Research Unit (SMRU), PO Box 46, Mae Sot, Tak 63110, Thailand; 2 Institute of Biomedical Engineering, Department of Engineering Science, University of Oxford, Oxford, UK; 3 Nuffield Department of Obstetrics & Gynaecology, University of Oxford, Oxford, UK; 4 Mahidol-Oxford Tropical Medicine Research Unit (MORU), Mahidol University, Bangkok, Thailand; 5 Centre for Clinical Vaccinology and Tropical Medicine, University of Oxford, Oxford, UK

**Keywords:** Malaria, Pregnancy, Three-dimensional ultrasound, Placenta volume, IUGR

## Abstract

**Background:**

The presence of malaria parasites and histopathological changes in the placenta are associated with a reduction in birth weight, principally due to intrauterine growth restriction. The aim of this study was to examine the feasibility of studying early pregnancy placental volumes using three-dimensional (3D) ultrasound in a malaria endemic area, as a small volume in the second trimester may be an indicator of intra-uterine growth restriction and placental insufficiency.

**Methods:**

Placenta volumes were acquired using a portable ultrasound machine and a 3D ultrasound transducer and estimated using the Virtual Organ Computer-aided AnaLysis (VOCAL) image analysis software package. Intra-observer reliability and limits of agreement of the placenta volume measurements were calculated. Polynomial regression models for the mean and standard deviation as a function of gestational age for the placental volumes of uninfected women were created and tested. Based on these equations each measurement was converted into a z -score. The z-scores of the placental volumes of malaria infected and uninfected women were then compared.

**Results:**

Eighty-four women (uninfected = 65; infected = 19) with a posterior placenta delivered congenitally normal, live born, single babies. The mean placental volumes in the uninfected women were modeled to fit 5th, 10th, 50th, 90th and 95th centiles for 14-24 weeks' gestation. Most placenta volumes in the infected women were below the 50th centile for gestational age; most of those with *Plasmodium falciparum *were below the 10th centile. The 95% intra-observer limits of agreement for first and second measurements were ± 37.0 mL and ± 25.4 mL at 30 degrees and 15 degrees rotation respectively.

**Conclusion:**

The new technique of 3D ultrasound volumetry of the placenta may be useful to improve our understanding of the pathophysiological constraints on foetal growth caused by malaria infection in early pregnancy.

## Background

Falciparum and vivax malaria have a markedly negative impact on mothers and babies [1,2]. For example, malaria infection in pregnancy is associated with a reduction in birth weight, principally due to intrauterine growth restriction (IUGR)[3,4]. Although the mechanisms responsible for IUGR are not fully understood, histopathological changes, such as thickening of basal membranes of the placenta[5], intervillositis[6-9], hypoxia[10,11] and syncytial destruction [12,13] have all been implicated.

The introduction of three-dimensional (3D) ultrasound has made it possible to assess intrauterine growth more accurately by allowing foetal organ and placental volumes to be measured [14,15]. For example, a small placental volume in the second trimester may be an indicator of IUGR and placental insufficiency [16,17]. Virtual Organ Computer-aided AnaLysis (VOCAL™, General Electric (GE) Healthcare, Austria) is a software analysis package used for calculating volumetry of different organs, such as the placenta [18], foetal volume [19] and organs including the foetal brain [20], thigh [21], spleen [22] and lungs [23].

This relatively new technique has never been used to study placenta volumes in pregnancies complicated by malaria. The aim of this study was to examine the feasibility of studying early pregnancy placental volumes in a malaria endemic area.

## Methods

The participants in the present study were attending the antenatal clinic (ANC) at Shoklo Malaria Research Unit (SMRU), which is located on the Thai-Burmese border. SMRU has focused on the epidemiology, prevention and treatment of malaria in pregnancy since 1986. The epidemiology of *Plasmodium (P.) falciparum *[24] and *P. vivax *[25] malaria in pregnancy is well described. There is a lack of effective prevention strategies and the malaria parasites are multi-drug resistant. Hence, SMRU runs an ANC programme with weekly screening to detect and treat all parasitaemic episodes during pregnancy to prevent maternal deaths [24]. All women are encouraged to attend the ANC as early as possible in pregnancy and to deliver at SMRU under the care of Advance Life Support in Obstetrics (ALSO) trained midwives and doctors; those requiring Caesarean section are transferred to the nearest Thai hospital.

The study was part of a larger foetal growth project (ClinicalTrials.gov Identifier: NCT00840502), approved by the Ethics Committees of Oxford (OxTREC (14-08)) and Mahidol (TMEC 2008-028) Universities.

### Ultrasonography

Ultrasound scans were performed trans-abdominally using a Voluson *i *(GE Healthcare, Austria) with a RAB2-5-RS; 2-5 MHz/Real time 3D probe. The machine was housed in a dedicated air-conditioned room equipped with a voltage stabilizer. All scans were obtained by sonographers specifically trained in foetal growth scanning [26], with regular internal quality control at SMRU; in addition, images were regularly sent for external quality control to the INTERGROWTH-21^st ^Project team at the University of Oxford [27].

Only women with singleton pregnancies were recruited. As the last menstrual period is largely unknown in this population [26], gestational age (GA) was determined by measuring the foetal Crown Rump Length (CRL) between 9^+0 ^and 13^+6 ^weeks. The protocol and operating manual for obtaining CRL measurements were similar to those used in the International Fetal and Newborn Growth Consortium for the 21st Century (INTERGROWTH-21st) Project[27]. Briefly, using three separate images, three blinded measurements were taken of each CRL; the mean of the three values was then used to estimate GA using Robinson's charts [28].

Thereafter, women were invited to attend for a foetal growth scan every 5 weeks until delivery to take standard 2D foetal biometric measurements. If a woman did not attend a scheduled scan then she was scanned at the next available opportunity. Every woman with a malaria episode detected by peripheral smear was treated using WHO protocols [29] and had foetal growth scans (in addition to those planned) at the time of a positive smear and 2 weeks later.

After standard 2D measurements were taken, a 3D sweep was obtained through the placenta. The 3D placental volume was acquired keeping the probe perpendicular to the placental plate and the size of the volume box was adapted to include the entire placenta. The sweep angle was set at 85° so as to visualize the maximum amount of placenta possible. Care was taken to minimize movement artifact. Once the scan was complete, volume data were stored on the system's hard drive for later analysis.

### Analysis

Only 3D ultrasound images of the placenta on the posterior uterine wall taken between 14^+0 ^to 23^+6 ^weeks (two sets of five weeks) were analysed. This approach was chosen because most placentas (especially on the anterior wall) are too large to fit in a single 3D sweep after 20 weeks. Based on published recommendations for cross-sectional studies, only one measurement per woman was included in the analysis [30,31]; if more than one good quality volume scan was available, the last one was analysed. Women who miscarried, and those with early onset pre-eclampsia or who left the study area before birth, were excluded. Scans were excluded from the analysis if the image quality was poor or a single continuous outline could not be drawn around the placenta as accurate volume data could not be obtained in these circumstances.

One author (WEM), blinded to clinical data, performed the volume measurements using the VOCAL™ method as described by Wegrzyn et al [32]. A sequence of sections of the placenta was obtained with the VOCAL™ method, which is based on the rotation of an object along its axis, using a predefined angle. Each placental volume was calculated with fixed angles of 30° (six sections) and 15° (12 sections). All volume measurements were taken twice for both rotation angles and an average of the 2 volume measurements was computed for each rotation to be used in the analysis.

Intra-observer reliability and limits of agreement (mean difference ± 1.96 SD) of the placenta volume measurements were calculated as described by Bland and Altman [33]. Systemic bias was determined by calculating the 95% confidence intervals (CI) for the mean difference between the measurements. If zero was included within this interval, no bias was assumed.

From the 15° measurements, separate polynomial regression models were fitted for the mean and standard deviation (SD), each as a function of GA [30], of the placenta volumes of women who had not been infected with malaria prior to the volume scan. This approach assumes that the distribution of measurements at each GA is Gaussian. The models were examined under the family of fractional polynomials utilizing its wide range of possibilities and flexibility [34]. An iterative procedure implemented in STATA version 11 statistical software (StataCorp, TX, USA) was used, whereby the model for the mean is weighted by the reciprocal of the square of the fitted SD [35]. A goodness-of-fit test of the models was also assessed by a likelihood ratio test by comparing the deviances. The estimated equations for the mean and the SD of these placenta volumes were of the form y = a + b*GA (where GA is in days). From these predictive mean and SD equations the corresponding centiles were calculated using the formula:

centile = mean + K * SD

where K is the corresponding centile from the theoretical Gaussian distribution (e.g. determination of the 10th and the 90th centiles requires K to be ± 1.28, and ± 1.645 for the fifth and 95th centiles).

Based on these equations the placental measurements of all women were converted into a z-score:

z - score = measured volume - expected volume / SD volume.

The advantage of using z-scores is that it eliminates the variability of measurements by GA, which allows for direct comparability of measurements. A negative z-score denotes a placental volume that is smaller than the expected mean for the population at a given GA. The z-scores of women who were or were not infected with malaria before the scan were compared.

Clinical data were entered using Microsoft Access, and analysed using SPSS version 15.0 for Windows (SPSS Inc., Chicago Ill, USA) and STATA version 11. The student's t-test was used to compare means, the Shapiro-Wilk test-to-test normality.

## Results

**Figure 1:**
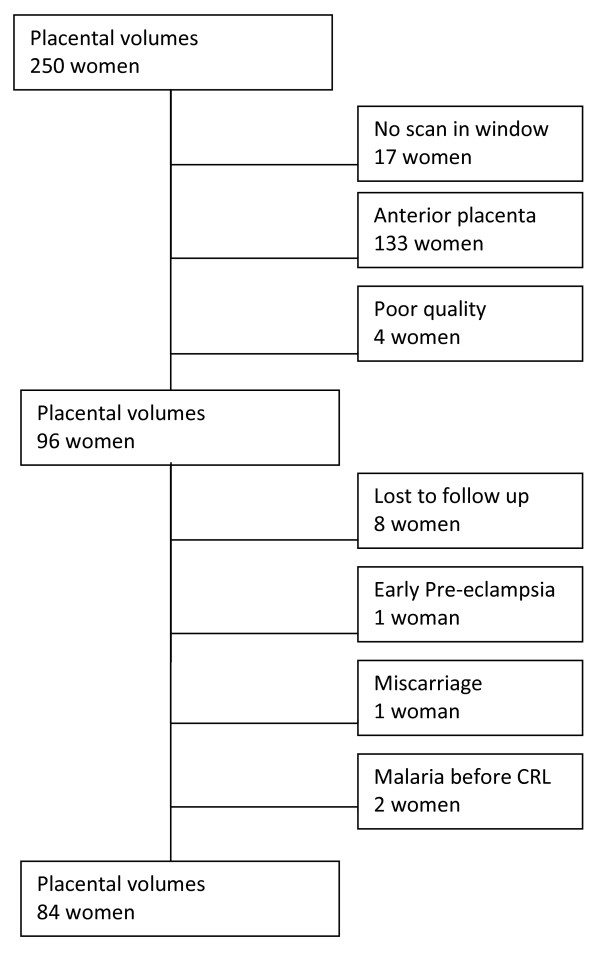
**Inclusion of placental scans**.

Between 10 February 2009 and 30 April 2010, a total of 250 women were recruited at a mean gestational age of 80 ± 10 (SD) days. Of these women, 84 (33.6%) were eligible for placental volume analysis as defined in the Methods section (Figure 1). Their baseline characteristics, e.g. age, parity, smoking status, etc., did not differ from the non-included women (data not shown). Of these 84 women, 19 (22.6%) had a malaria episode before the volume scan; the other 65 women remained malaria free (Table 1). All women delivered congenitally normal, live born, single babies.

**Table 1:** Baseline characteristics of women

	Malaria (N = 19)	Non malaria (N = 65)
Age (years)	25.1 ± 5.3	27.6 ± 7.0

Teenager, n (%)	3 (16)	7 (11)

Gravida	2 [1-7]	3 [1-14]

Parity	1 [0-5]	1 [0-13]

Primigravida	6 (32)	25 (29)

Smoking	4 (21)	11 (17)

Height (cm)	152 ± 5	153 ± 4

Hct at time of ultrasound (%)	31.2 ± 2.7	32.6 ± 2.8

HBP or PE	3 (16)	5 (8)

Non-malarial infection	3 (16)*	6 (9)#

Data are presented as median [range], mean ± SD, or n (%)

HBP high blood pressure, Hct Haematocrit, PE pre-eclampsia, SD standard deviation

* dysentery (32 weeks), pneumonia (24 weeks), suspected scrub typhus (8 weeks)

# pneumonia (39 weeks), pyelonephritis (20 weeks), suspected scrub typhus (3 cases; 25, 26 and 40 weeks), unspecified sepsis (36 weeks)

### Measurement variability

**Figure 2:**
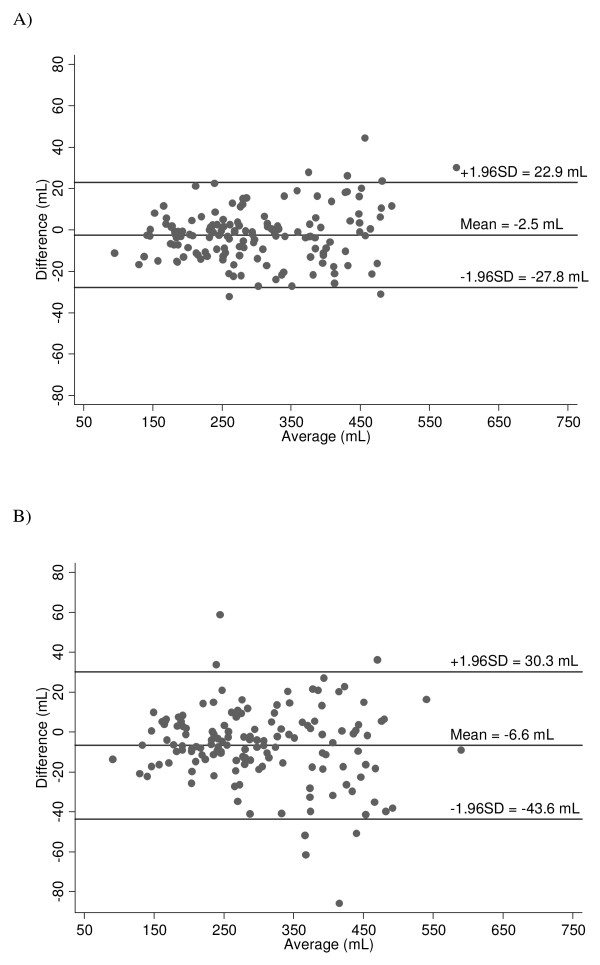
**Intra-observer variability of placental volume measurements**. Plot of difference against mean for intra-observer variability of 145 placental volume measurements in 96 women using VOCAL ™ at 15° (A) and 30° (B) rotational angles, with mean difference and 95% limits of agreement indicated.

For this part of the analysis the volume of all placentas (n = 96 women, see Figure 1) were included irrespective of birth outcome or malaria status. The difference between the first and second measurements and the different rotation angles on the same 3D volume were normally distributed. There was no significant difference between the mean placental volumes for a single measurement at 30° and 15°, *p *= 0.27. The mean difference between the two measurements was 6.6 (95% CI: 3.6-9.70) mL at 30°, and 2.5 (95% CI: 0.3-4.6) mL at 15°. The 95% intra-observer limits of agreement for first and second measurements were ± 37.0 mL at 30° and ± 25.4 mL at 15° (Figure 2).

### Placental volumes in uninfected women

The fractional polynomial linear regression model yielded the following formulae:

a) Mean volume = - 162.83 + 3.60 × GA

b) SD = - 41.55 + 0.80 × GA

A simple linear model with no quadratic or cubic terms for both the mean and SD provided the best fit for the model with a deviance of 717.98. The z-scores from the model did not suggest any deviation from normality using the Shapiro-Wilk test, *p *= 0.36.

### Malaria

**Figure 3:**
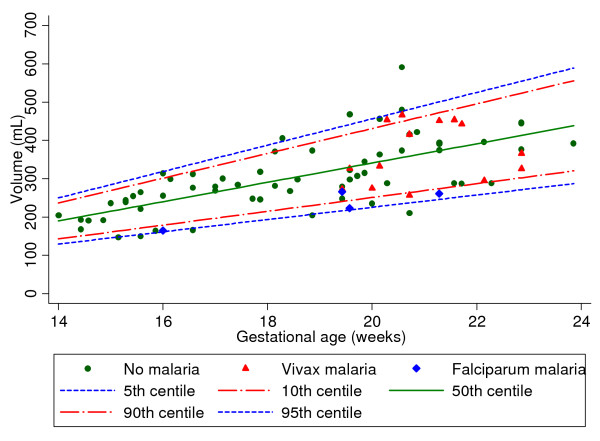
**Placental volume centiles with placental volumes of women with falciparum and vivax malaria. **Fitted the fifth and 95th (blue lines), 10th and 90th (red lines) and 50th (green line) centile curves on the placental volumes of women without malaria or adverse birth outcomes (green dots) and superimposed the placental volumes of women with *Plasmodium falciparum *malaria (blue diamonds) or *Plasmodium vivax *malaria (red triangles).

Nineteen women were infected with malaria between the dating and placental volume scans: four (21%) with *P. falciparum *and 15 (79%) with *P. vivax*. The baseline characteristics of infected and uninfected women were similar, except for a lower mean haematocrit at the time of the volume scan, as expected following infection with malaria: 31.2 ± 2.7% *versus *32.6 ± 2.8% respectively, *p *= 0.05 (Table 1). The median number of malaria episodes was one [range 1-4]. Four women had more than one malaria episode in this period: three women had multiple *P. vivax *episodes (four, two and two episodes), and one woman had one *P. vivax *and one *P. falciparum *episode and was classified as *P. falciparum*. The median time between the last episode of malaria and the volume scan was 29 (IQR 14-42, range 0-59) days and this was not different for the *P. falciparum *malaria or *P. vivax *malaria group. Figure 3 illustrates the model fit to the raw data for each measurement with the fifth, 10th, 50th, 90th and 95th centiles for placenta volumes between 14^+0 ^and 23^+6 ^weeks obtained from the uninfected women (green dots). Most of the placental volumes of the infected women as a whole were below the 50th centile; most of those with *P. falciparum *were below the 10th centile (Figure 3).

### Placental volume z-scores

The mean z-scores were similar in women when compared for smoking status, gravidity, age, anaemia (haematocrit < 30%) and those who had other infections in pregnancy (Table 2). However, the difference was significant for women with *P. falciparum *infections, *p *= 0.003 (Table 2 and Figure 3). The z-scores could not be related to the number of malaria episodes or symptoms because of the small numbers involved.

**Table 2:** Risk factors associated with placental volume: z-scores

		N (%)	Z-score Mean ± SD	*P*-value
Smoking	Yes	15 (18)	0.00 ± 1.1	0.73
	
	No	69 (82)	-0.11 ± 1.0	

Primigravida	Yes	31 (37)	-0.14 ± 1.1	0.72
	
	No	53 (63)	-0.06 ± 1.0	

Non-malarial infection	Yes	9 (11)	-0.14 ± 0.55	0.80
	
	No	75 (89)	-0.08 ± 1.1	

Anaemia	Yes	16 (19)	-0.38 ± 0.86	0.16
	
	No	68 (81)	-0.02 ± 1.1	

Teenager	Yes	10 (12)	0.09 ± 1.5	0.70
	
	No	74 (88)	-0.11 ± 1.0	

Malaria*	Yes	19 (23)	-0.38 ± 1.0	0.17
	
	No	65 (77)	0.00 ± 1.0	

P. falciparum*	Yes	4 (6)	-1.30 ± 0.4	**0.003**
	
	No	65 (94)	0.00 ± 1.0	

P. vivax*	Yes	15 (19)	-0.13 ± 1.0	0.66
	
	No	65 (81)	0.00 ± 1.0	

*occurring between the CRL dating and volume scans

## Discussion

This pilot study demonstrates the feasibility of a) relating placental volume to gestational age between 14 and 24 weeks in a malaria endemic area, and b) comparing volumes between women with and without malaria. The findings are important because measurement of placental volume in the first or second trimester may predict which pregnancies are at high-risk of adverse outcomes [17,36-38].

A variety of 2D and 3D ultrasound methods to measure placental volume have been reported [32,36,39,41] but, in clinical practice, 3D may be more accurate than 2D methods [36]. VOCAL™, one commercially available method of analysing 3D images, is considered relatively fast and reproducible. It allows the borders of the target organ to be modified after volumetric calculations and it is superior to other methods in evaluating very irregularly shaped structures, such as the placenta [42]. However, accurate 3D volume measurement of the whole placenta is only possible in the first half of pregnancy because the volume seen is limited by the transducer footprint [36].

Accuracy is also influenced by the rotation angle of the analysis method. In this study, a small error was observed between the volume measurements when comparing the two rotation methods, 30° and 15° (Figure 2). The 30° rotation method resulted in a wider range of intra-observer 95% limits of agreement than the 15° method. Differing reproducibility data have been reported for 3D measurements and analyses of placental volume, varying from relatively poor to highly similar intra-and inter-observer agreement [39,42-46]]. The results of this study are similar to those of Cheong et al, who reported that measurements made with VOCAL 30°, in an ex-vivo experiment, were faster to complete, but associated with significantly higher variability than those made with VOCAL 12° [18].

As in studies of foetal organ volumetry [19], placental studies show wide discrepancies in reference ranges [36]. The volumes reported in this study seem larger than previously published data [17,36,38,40,41,45]. Rather than indicating true biological differences between populations, the discrepancies are more likely due to methodological differences; for example, in defining the placental border. Hence, there is a clear need to standardize 3D volumetric methods, and definitions of imaging planes and anatomical landmarks in particular. In the absence of standardized methods, this dataset was not compared with previously published placental volumes; rather, the focus was on studying the effect of malaria within the same population using a single set of well-defined methods.

In this preliminary investigation, infection with *P. falciparum *before 24 weeks' gestation appears to be associated with smaller placental volumes. In other studies, principally in developed countries, early placental volumetry has been shown to predict IUGR and adverse pregnancy outcomes [47,48], due probably to impaired trophoblast invasion; in addition, small placentas in the first trimester are associated with high resistance uterine perfusion in the second trimester [49]. All these factors have previously been related to malaria in pregnancy as well [1,50,51].

Infection with *P. vivax *did not seem to be related to placenta volume. The mechanisms underlying the adverse effects of *P. vivax *malaria in pregnancy are not fully understood [25,52,53]. Systemic or hormonal mechanisms may play a role in *P. vivax*-related growth restriction, as there is very little evidence that *P. vivax *sequesters in the placenta, as *P*. *falciparum *does [53].

There was a borderline difference in haematocrit at the time of the placental volume scan between the groups (Table 1), however anaemia was not related to placenta volume in the logistic regression model (Table 2). The timing of anaemia in pregnancy and the effect on placental weight and volume [1] needs further investigation.

This study, therefore, suggests that 3D placental volumetry is worthy of further investigation, in order to assess whether IUGR related to malaria is mediated via a smaller placental volume.

The data presented here involve small numbers and should be interpreted cautiously. Another limitation of the study was the initial decision to include only placentas on the posterior wall to maximize the likelihood of capturing the whole target organ in the volume sweep. This enabled the inclusion of complete placental volumes up until 24 weeks' gestation, but resulted in a large group of women with an anterior placenta being excluded (n = 133, Figure 1). The data used in this study may not be comparable with other populations because of the methodological differences described above. Lastly, no inter-observer variability analysis was available [19] but, for the purpose of this analysis, which compared volumes measured by a single observer using the same methods, this was not a vital constraint.

Generally, ultrasound machines with 3D measurement capacity are delicate, expensive, and require a high level of technical skills and are therefore not available in most malaria endemic areas. Nevertheless, the suggestion that malaria in early pregnancy reduces placental volume in the second trimester should be confirmed by prospective studies evaluating volumes in relation to foetal growth and adverse pregnancy outcomes. Such studies should also allow for better assessment of potential confounders (such as maternal anaemia [1,54], smoking [55] and parental anthropometry [56]). These are essential steps in unravelling the sequence of events from maternal malaria infection to IUGR.

In conclusion, the new technique of 3D ultrasound volumetry of the placenta may be useful to improve our understanding of the pathophysiological constraints on foetal growth caused by malaria infection in early pregnancy.

## Competing interests

The authors declare that they have no competing interests.

## Authors' contributions

MJR designed the study, carried out the ultrasound scanning, performed the statistical analysis and drafted the manuscript. WEM performed the volume measurements, helped with the statistical analysis and drafted the manuscript. SK carried out the ultrasound scanning and organized the study on site. GS helped with the volume measurements and organized the database. EO performed the statistical analysis and helped to draft the manuscript. JAN participated in the design and coordination of the study. SHK participated in its design and coordination of the study. RMG participated in the design and coordination of the study, helped in the statistical analysis and helped to draft the manuscript. ATP participated in the design and coordination of the study, helped in the statistical analysis and helped to draft the manuscript. FN conceived of the study and participated in the design and coordination of the study, and helped to draft the manuscript. All authors read and approved the final manuscript.
